# Association of OCT-Derived Drusen Measurements with AMD-Associated Genotypic SNPs in the Amish Population

**DOI:** 10.3390/jcm4020304

**Published:** 2015-02-12

**Authors:** Venkata Ramana Murthy Chavali, Bruno Diniz, Jiayan Huang, Gui-Shuang Ying, SriniVas R. Sadda, Dwight Stambolian

**Affiliations:** 1 Department of Ophthalmology, University of Pennsylvania, 313B Stellar-Chance Labs, 422 Curie Blvd., Philadelphia, PA 19104, USA; E-Mail: vchavali@mail.med.upenn.edu; 2 Doheny Eye Institute, Los Angeles, CA 90033, USA; E-Mails: bdinizlt@hotmail.com (B.D.); vassadda@gmail.com (S.R.S.); 3 Department of Ophthalmology, Universidade Federal de São Paulo, São Paulo 09920, Brazil; 4 Center for Preventive Ophthalmology and Biostatistics, Department of Ophthalmology, University of Pennsylvania, Philadelphia, PA 19104, USA; E-Mails: huangjiayan@gmail.com (J.H.); gsying@mail.med.upenn.edu (G.-S.Y.); 5 Department of Ophthalmology, Keck School of Medicine of the University of Southern California, Los Angeles, CA 90033, USA

**Keywords:** age-related macular degeneration, AMD, Older Order Amish, CFH, SYN3, OCT, drusen area, drusen volume, RPE atrophy, Cirrus HD-OCT

## Abstract

Purpose: To investigate the association of optical coherence tomography (OCT)-derived drusen measures in Amish age-related macular degeneration (AMD) patients with known loci for macular degeneration. Methods: Members of the Old Order Amish community in Pennsylvania ages 50 and older were assessed for drusen area, volume and regions of retinal pigment epithelium (RPE) atrophy using a Cirrus High-Definition OCT. Measurements were obtained in the macula region within a central circle (CC) of 3 mm in diameter and a surrounding perifoveal ring (PR) of 3 to 5 mm in diameter using the Cirrus OCT RPE analysis software. Other demographic information, including age, gender and smoking status, were collected. Study subjects were further genotyped to determine their risk for the AMD-associated SNPs in the *SYN3*, *LIPC*, *ARMS2*, *C3*, *CFB*, *CETP*, *CFI* and *CFH* genes using TaqMan genotyping assays. The association of genotypes with OCT measures were assessed using linear trend *p*-values calculated from univariate and multivariate generalized linear models. Results: 432 eyes were included in the analysis. Multivariate analysis (adjusted by age, gender and smoking status) confirmed the known significant association between AMD and macular drusen with the number of CFH risk alleles for the drusen area (the area increased 0.12 mm^2^ for a risk allele increase, *p* < 0.01), drusen volume (the volume increased 0.01 mm^3^ for a risk allele increase, *p* ≤ 0.05) and the area of RPE atrophy (the area increased 0.43 mm^2^ for a risk allele increase, *p* = 0.003). *SYN3* risk allele G is significantly associated with larger area PR (the area increased 0.09 mm^2^ for a risk allele increase, *p* = 0.03) and larger drusen volume in the central circle (the volume increased 0.01 mm^3^ for a risk allele increase, *p* = 0.04). Conclusion: Among the genotyped SNPs tested, the CFH risk genotype appears to play a major role in determining the drusen phenotype in the Amish AMD population.

## 1. Introduction

Age-related macular degeneration (AMD) is a leading cause of vision loss and is influenced by genetic, environmental and dietary factors [1]. It is estimated that in the United States alone, the number of advanced AMD cases may reach up to three million by the year 2020 [2]. Clinical features of AMD include the death of photoreceptors and retinal pigment epithelium (RPE). Drusen are hallmark deposits associated with early AMD and considered to be a major risk factor for the progression of AMD [3,4,5]. The late stages of AMD are classically divided into two forms: non-neovascular (“dry”) AMD/atrophic AMD and neovascular (“wet”) AMD. Several studies have identified a variety of potential risk factors associated with AMD [6].

In recent years, significant advances have been made in our understanding of the genetic basis of AMD. Gene variants or single-nucleotide polymorphisms (SNPs) are routinely used to test the association with disease phenotypes. SNPs that increase risk or are protective have been identified for several complement-related genes: factor H (CFH) [7,8,9,10], the complement factor B region (CFB) [11,12], complement factor I [13], complement C3 [14,15] and the ARMS2/serine protease HTRA1 [16,17,18,19]. A variant in the hepatic lipase gene, *LIPC*, was reported to decrease the risk of advanced AMD in a genome-wide association study (GWAS) [20,21]. Other genes in the HDL pathway and cholesterol transport, including CETP, are hypothesized to be involved in the etiology of AMD [22].

The risk of AMD progression has been positively correlated with total drusen area and the drusen size using fundus photography [23,24]. Color fundus photographs (CFPs) are the most widely-used method for the assessment of AMD. However, reliable quantitation of drusen using CFPs is challenging due to the macular background from RPE and choroidal pigmentation [25]. Furthermore, most published methods for grading CFPs for drusen feature a qualitative or semi-quantitative approach and are thus less sensitive to small changes in drusen number in early disease. Furthermore, as CFPs are two-dimensional, only the drusen area can be estimated, while drusen thickness and volume cannot be measured. We had earlier developed an automated drusen detection system for classifying age-related macular degeneration (AMD) from color fundus photographs (CFPs) to overcome a few of these limitations [26]. More recently, optical coherence tomography (OCT) has been advocated as a potentially useful technique for *in vivo* cross-sectional imaging of drusen and quantitative evaluation of retinal structure. Development of higher speed and higher sensitivity spectral domain OCT systems (SD-OCT) has further enhanced our ability to obtain high resolution imaging of the RPE and drusen [27,28].

In the current study, we describe the correlation of OCT-derived measures of drusen and RPE atrophy in an elderly Amish population with SNP genotypes in the *CFH*, *CFI*, *CFB*, *CETP*, *C3*, *ARMS2*, *LIPC* and *SYN3* genes.

## 2. Methods

### 2.1. Subjects

Amish individuals living in Lancaster County, Pennsylvania, with early AMD were selected for inclusion in this study. These individuals were discovered as part of a larger population-based study in the Old Order Amish. All subjects were given a complete eye examination with pupillary dilation.

All of the study participants, aged 50 years and older, were asked to complete a brief questionnaire about present or past cigarette smoking history and the number of years of smoking. Simultaneously, 10 mL of blood were drawn from each patient by a certified phlebotomist. Digital stereo images of the macula, optic disc and macular OCT scans were obtained from all study participants using a Zeiss Cirrus OCT 4000 (Carl Zeiss Meditec, Dublin, CA, USA). The study was approved by the Institutional Review Boards of the University of Pennsylvania and University of Southern California and is consistent with the tenets set forth in the Declaration of Helsinki.

### 2.2. Genotyping

DNA was extracted from peripheral blood leukocytes using standard protocols (QiaAmp DNA Blood Mini kit, Cat#51106). We genotyped a total of 8 single-nucleotide polymorphisms for the following SNPs: *SYN3* (rs5749482), *LIPC* (rs920915), *ARMS2/HTRA1* (rs10490924), *C3* (rs2230199), *CFB* (rs429608), *CETP* (rs3764261), *CFI* (rs10033900) and *CFH* (rs12038333) genes. The genotyping was done using TaqMan SNP Genotyping assays (Life Technologies, Grand Island, NY, USA) following the manufacturer’s instructions. Briefly, all PCR amplifications were performed with the following thermal cycling conditions: 95 °C for 10 min followed by 40 cycles of 95 °C for 15 s and 60 °C for 1 min. PCR reactions were performed with TaqMan Genotyping Master Mix (Life Technologies, NY, USA) on a 7900HT Fast Real-Time PCR System (Life Technologies, NY, USA). All pre- and post-PCR plate readings were performed on a 7900HT Fast Real-Time PCR System, and the allele types were confirmed by the system’s software (7900HT Fast Real-Time PCR System SDS software version 2.3; Life Technologies).

### 2.3. Optical Coherence Tomography Acquisition

OCTs were obtained using the Cirrus OCT 4000 with a raster scan protocol (200 × 200). This acquisition protocol has been well established for gathering quantitative measurements of drusen area and drusen volume [28]. Scans of each eye were obtained after dilation and covered a retinal area of 6 × 6 mm, centered on the fovea. After acquisition, scans were immediately assessed by the operator for image quality (signal strength >7) and motion artifacts, and scans were repeated multiple times if necessary in order to obtain the best possible quality scan for subsequent analysis.

### 2.4. Quantification of Drusen and Areas of RPE Atrophy

Drusen and RPE analyses were performed using the FDA-cleared Cirrus Version 6.0 Advanced RPE Analysis algorithm. The reproducibility of this algorithm has been previously published [29,30,31], and we have demonstrated good agreement with manual segmentation of drusen by expert reading center graders [28,29]. The algorithm computes drusen area and volume by estimating the original RPE floor and subtracting it from the detected RPE surface (elevated by the drusen) [28]. The drusen area and volume are reported within a 3-mm diameter circle centered on the fovea, as well as within a surrounding perifoveal ring (outer ring diameter of 5 mm). Areas of RPE atrophy are also detected and quantified (within the 6 × 6-mm scanning square) automatically by the software based on identification of increased transmission of light into the choroid.

Two hundred sixty eight subjects were recruited for this study, but 51 subjects were excluded from subsequent quantitative analysis due to poor quality scans, missing scans or features of neovascular AMD, such as sub-retinal fluid, intra-retinal edema, sub-retinal tissue or serous pigment epithelial detachments in the OCT B-scans in at least one eye. One subject was excluded due to poor genotyping quality. Thus, 432 eyes of 216 consecutive subjects were included in this analysis. As one of our overall study objectives was to evaluate the correlation between eyes [32], only subjects with good quality scans for both eyes were considered for subsequent analysis.

### 2.5. Statistical Methods

To determine if there was an association among 3 genotypes of each SNP and drusen area/volume or area of RPE atrophy, the number of risk alleles for each genotype was counted as either 0, 1 or 2, and genotype association with each OCT measure from an eye was evaluated using tests of linear trend from generalized linear models with inter-eye correlation accounted for using generalized estimating equations (GEE). For the SNP of SYN3, only two genotypes (GG and CG) were observed in the study participants, and the comparison between two genotypes were performed using analysis of variance, with inter-eye correlation accounted for using GEE. The effect for each risk allele on the drusen area/volume was estimated based on the slope of generalized linear regression models. These evaluations were evaluated using univariate analysis (without adjustment of risk factors of AMD) and multivariate analysis (with adjustment of age, gender and smoking status). To account for multiple comparisons from multiple SNPs and multiple OCT measurements, we adjusted *p*-values using the global false discovery rate [33]. All analyses were performed in SAS V9.2 (SAS Institute Inc., Cary, NC, USA) software; *p* < 0.05 was considered to be statistically significant.

## 3. Results

A total of 268 subjects were recruited for our study; 51 subjects were excluded either due to poor quality scans or features of neovascular AMD on OCT in at least one eye, and one additional subject was removed due to poor quality genotypes. Thus, 432 eyes of 216 subjects were included for analysis. SNP genotyping for eight SNPs was completed on all 216 subjects. Our sample included a total of 122 women and 94 men with a mean age (±standard deviation) of 73 ± 6.6 years (range: 50–89). The majority of the subjects was between 70 and 79 years of age (56.1%), and 50 (23.2%) had a history of smoking (Table 1).

**Table 1:** Characteristics of study subjects (*N* = 216).

**Age (years)**	
50–59	5 (2.3%)
60–69	57 (26.4%)
70–79	120 (55.6%)
≥80	32 (14.8%)
Unknown	2 (0.9%)
Mean (standard deviation)	70.3 (6.63)
**Gender**	
Female	122 (56.5%)
Male	94 (43.5%)
**Ever Smoked**	
Yes	50 (23.2%)
No	157 (72.7%)
Unknown	9 (4.2%)

Table 2 shows the comparison of drusen areas/volumes and areas of RPE atrophy with genotype. In univariate analysis, significant associations of the drusen area in the perifoveal ring (PR, *p* = 0.006) and drusen area in the central circle (CC, *p* = 0.02) were observed for the risk allele GG in *SYN3* (SNP, rs5749482). The drusen volume CC (*p* = 0.02) and drusen volume PR (*p* = 0.03) were also significantly associated with *SYN3*. The association of the area of RPE atrophy with *SYN*3 showed a trend, but did not reach statistical significance (*p* = 0.07). The associations between drusen area PR (*p* = 0.03) and drusen volume CC (*p* = 0.04) with *SYN*3 remained even after adjustment for age, gender and smoking status. However, these associations become non-significant after multiple comparison adjustment for the total of 40 tests in this study. It is interesting to observe that the majority of the 216 subjects have the *SYN3* GG risk alleles (91%). This high percentage of GG risk alleles could be attributed to interbreeding and being isolated in terms of lifestyle and culture. The homozygous CC genotype was not observed in our study population.

In univariate analysis, we observed a significant association of the CFH risk allele GG for SNP, rs12038333, with drusen area CC (*p* = 0.007), drusen area PR (*p* = 0.002), drusen volume CC (*p* = 0.049), drusen volume PR (*p* = 0.002) and area of RPE atrophy (*p* = 0.003); and these associations remained after adjustment for age, gender and smoking status (Table 2). Even after multiple comparison adjustment, the association between CFH with drusen area PR and drusen volume CC (*p* = 0.04, respectively, Table 2) remained significant. Our results indicate that SNPs in the *CFH* and *SYN3* genes are important influences for drusen size and volume in the macula and perifoveal region, a hallmark of AMD.

The drusen area, drusen volume and regions of RPE atrophy did not show any significant association with SNPs in the other genes that were tested (Table 2).

**Table 2:** Genotypic associations with optical coherence tomography (OCT) drusen area, drusen volume and area of retinal pigment epithelium (RPE) atrophy from univariate analysis and multivariate analysis ^§^ (216 subjects, 432 eyes).

SNP	Genotype	*N*	Drusen Area in mm^2^: Mean (SE)		Drusen Volume in mm^3^: Mean (SE)		Area of RPE Atrophy in mm^2^: Mean (SE)
			Central Circle	Perifoveal Ring	Central Circle	Perifoveal Ring	
normal/(risk) SYN3 C/(G) rs5749482	GG	198	0.23 (0.05)	0.18 (0.04)	0.02 (0.01)	0.008 (0.002)	0.50 (0.11)
	CG	18	0.06 (0.04)	0.03 (0.02)	0.002 (0.002)	0.002 (0.001)	0.19 (0.11)
	CC	0	-	-	-	-	-
	Univariate *P* *		0.02	0.006	0.02	0.03	0.07
	Adjusted Slope (SE) ^†^		0.11 (0.06)	0.09 (0.04)	0.01 (0.005)	0.004 (0.002)	0.09 (0.16)
	Multivariate *P*^†^		0.08	0.03	0.04	0.09	0.58
	Multiple comparison adjusted *P* by global FDR ^‡^		0.36	0.26	0.26	0.36	0.84
(risk)/normal LIPC (C)/G rs920915	CC	58	0.26 (0.08)	0.24 (0.08)	0.02 (0.01)	0.01 (0.01)	0.78 (0.29)
	CG	122	0.22 (0.07)	0.15 (0.04)	0.02 (0.01)	0.007 (0.002)	0.40 (0.11)
	GG	37	0.11 (0.03)	0.09 (0.04)	0.004 (0.001)	0.004 (0.002)	0.22 (0.09)
	Univariate Linear Trend *P* *		0.16	0.14	0.28	0.19	0.08
	Adjusted Slope (SE) ^†^		0.06 (0.05)	0.07 (0.04)	0.005 (0.004)	0.004 (0.003)	0.21 (0.13)
	Multivariate Linear Trend *P*^†^		0.16	0.14	0.26	0.20	0.12
	Multiple comparison adjusted *P* by global FDR ^‡^		0.49	0.47	0.69	0.57	0.44
normal/(risk) ARMS2 G/(T) rs10490924	TT	20	0.48 (0.14)	0.39 (0.10)	0.02 (0.01)	0.01 (0.00)	1.48 (0.55)
	GT	81	0.15 (0.05)	0.13 (0.05)	0.008 (0.003)	0.005 (0.002)	0.45 (0.18)
	GG	114	0.21 (0.07)	0.15 (0.05)	0.02 (0.01)	0.008 (0.003)	0.31 (0.10)
	Univariate Linear Trend *P* *		0.40	0.20	0.48	0.77	0.045
	Adjusted Slope (SE) ^†^		0.04 (0.07)	0.05 (0.05)	−0.01 (0.01)	−0.0003 (0.003)	0.37 (0.18)
	Multivariate Linear Trend *P*^†^		0.59	0.41	0.41	0.92	0.051
	Multiple comparison adjusted *P* by global FDR ^‡^		0.84	0.77	0.77	0.95	0.26
(risk)/normal C3 (C)/G rs2230199	CC	1	0.20 (0.00)	0.05 (0.00)	0.008 (0.000)	0.002 (0.000)	1.60 (0.00)
	CG	22	0.30 (0.13)	0.20 (0.10)	0.01 (0.01)	0.007 (0.004)	0.68 (0.43)
	GG	194	0.20 (0.05)	0.16 (0.04)	0.01 (0.01)	0.008 (0.002)	0.44 (0.10)
	Univariate Linear Trend *P* *		0.51	0.80	0.82	0.78	0.45
	Adjusted Slope (SE) ^†^		0.08 (0.12)	0.01 (0.09)	−0.001 (0.01)	−0.002 (0.004)	0.27 (0.37)
	Multivariate Linear Trend *P*^†^		0.50	0.91	0.91	0.66	0.48
	Multiple comparison adjusted *P* by global FDR ^‡^		0.77	0.95	0.95	0.88	0.77
normal/(risk) CFB A/(G) rs429608	GG	196	0.21 (0.04)	0.17(0.04)	0.01(0.00)	0.008(0.002)	0.49(0.11)
	AG	19	0.23 (0.20)	0.12(0.10)	0.03(0.02)	0.008(0.008)	0.33(0.21)
	AA	2	0.000 (0.000)	0.02 (0.02)	0.000 (0.000)	0.001 (0.001)	0.12 (0.09)
	Univariate Linear Trend *P* *		0.90	0.43	0.73	0.88	0.35
	Adjusted Slope (SE) ^†^		0.02 (0.14)	0.06 (0.08)	−0.01 (0.02)	0.001 (0.01)	0.13 (0.16)
	Multivariate Linear Trend *P*^†^		0.90	0.47	0.75	0.89	0.46
	Multiple comparison adjusted *P* by global FDR ^‡^		0.95	0.77	0.94	0.95	0.77
(risk)/normal CETP (A)/C rs3764261	AA	21	0.10 (0.06)	0.09 (0.05)	0.003 (0.002)	0.003 (0.002)	0.57 (0.36)
	AC	97	0.28 (0.07)	0.20 (0.06)	0.02 (0.01)	0.01 (0.00)	0.51 (0.16)
	CC	96	0.17 (0.06)	0.16 (0.05)	0.01 (0.01)	0.006 (0.002)	0.42 (0.14)
	Univariate Linear Trend *P* *		0.71	0.91	0.96	0.83	0.63
	Adjusted Slope (SE) ^†^		0.03 (0.05)	0.002 (0.04)	0.001 (0.01)	0.001 (0.002)	0.14 (0.16)
	Multivariate Linear Trend *P*^†^		0.62	0.97	0.93	0.72	0.37
	Multiple comparison adjusted *P* by global FDR ^‡^		0.86	0.97	0.95	0.93	0.77
normal/(risk) CFI C/(T) rs10033900	TT	17	0.14 (0.09)	0.06 (0.02)	0.01 (0.01)	0.002 (0.001)	0.32 (0.25)
	CT	115	0.19 (0.04)	0.18 (0.05)	0.010 (0.004)	0.008 (0.003)	0.55 (0.15)
	CC	85	0.26 (0.09)	0.17 (0.06)	0.02 (0.01)	0.009 (0.003)	0.39 (0.16)
	Univariate Linear Trend *P* *		0.36	0.51	0.30	0.44	0.72
	Adjusted Slope (SE) ^†^		−0.07 (0.07)	−0.03 (0.05)	−0.01 (0.01)	−0.002 (0.003)	0.03 (0.16)
	Multivariate Linear Trend *P*^†^		0.36	0.50	0.31	0.47	0.84
	Multiple comparison adjusted *P* by global FDR ^‡^		0.77	0.77	0.77	0.77	0.95
normal/(risk) CFH A/(G) rs12038333	GG	49	0.30 (0.08)	0.29 (0.08)	0.01 (0.00)	0.01 (0.00)	1.15 (0.32)
	AG	99	0.26 (0.08)	0.19 (0.06)	0.02 (0.01)	0.01 (0.00)	0.36 (0.13)
	AA	69	0.08 (0.04)	0.04(0.02)	0.005 (0.003)	0.002 (0.001)	0.14 (0.07)
	Univariate Linear Trend *P* *		0.007	0.002	0.049	0.002	0.003
	Adjusted Slope (SE) ^†^		0.11 (0.04)	0.12 (0.04)	0.01 (0.003)	0.005 (0.002)	0.43 (0.14)
	Multivariate Linear Trend *P* ^†^		0.008	0.001	0.050	0.002	0.004
	Multiple comparison adjusted *P* by global FDR ^‡^		0.08	0.04	0.26	0.04	0.053

^§^ Adjusted by age, gender and smoking status; * From univariate analysis (without adjustment by any other risk factors of age-related macular degeneration (AMD)) for testing whether OCT measures were associated with the number of risk alleles; ^†^ From multivariate analysis (with adjustment by age, gender and smoking status) for testing whether OCT measures were associated with the number of risk alleles; ^‡^ Adjusted using global FDR (false discovery rate) for multiple comparisons from eight SNPs and five OCT measurements (*i.e.*, adjustment for a total of 40 tests) for *p*-values from multivariate analysis.

## 4. Discussion

We have examined the association of eight known AMD genetic variants with drusen phenotypes assessed by the Cirrus OCT. There was a strong association between drusen area, volume and RPE atrophy in the macula with risk alleles in *CFH* (rs12038333) and *SYN3* (rs5749482). These eight SNPs have been reported to be highly associated with AMD [34].

The role of genetic variants in drusen accumulation and early AMD has received little attention [35,36,37]. HDL pathway genes are reported to be associated with the early stages of AMD, whereas ARMS2/HTRA1 and genes in the complement pathway are associated with more advanced stages [35]. CFH, ARMS2/HTRA1 and C3 genes have been reported to increase the risk of progression from intermediate drusen to large drusen and from large drusen to geographic atrophy and neovascularization. The T allele of rs1883025 in ABCA1 is associated with a decreased risk of intermediate drusen, large drusen, geographic atrophy (GA) and neovascularization [35], whereas the C allele of COL8A1 is protective of the transition from intermediate to large drusen [38].

Significant association of drusen progression with CFH has been reported in Age-Related Eye Disease Study (AREDS) and familial AMD cohorts [39]. Smoking is strongly associated with a 2.5- to 4.5-fold increased risk for late AMD [40], and its risk is known to increase 6–14-fold with the presence of the CFH polymorphism [41]. The CFH Y402H is reported to act synergistically with smoking to increase the risk of wet AMD [42]. We have observed a strong association of the CFH SNP rs1203833 with macular drusen area and volume in our Amish AMD cohort, thus supporting the hypothesis that this variant is relevant to drusen formation.

Variants in the *SYN3* gene are reported to influence susceptibility to AMD [43]. The *SYN3* gene is a member of the *Synapsin* gene family and is known to play a role in synaptogenesis and in the modulation of neurotransmitter release. The associated *SYN3* SNP (rs5749482) with AMD is located in the *SYN3* intron 6. This SNP could be influencing the expression of *SYN3* or a nearby gene, *TIMP3*, which is located within the same intron. Further screening of Amish cohorts with SNP, rs9621532, in TIMP3 could elucidate its role in AMD [43,44,45]. To our knowledge, this is the first report to demonstrate an association of *SYN3* with quantitative macular drusen burden in AMD. Additional SNPs for the *SYN3* gene need to be screened to further understand the role of this gene in influencing drusen formation.

Apart from *CFH* and *SYN3*, the other genotyped genes, *ARMS2*, *C3*, *CFB*, *LIPC*, *CETP* and *CFI* genes, were not associated with our drusen phenotypes. This could be due to insufficient power in our study to detect small effects. Additional studies of these SNPs in different populations are needed to establish haplotypes for drusen burden in AMD. Our study was limited to subjects older than 50 years. Therefore, we cannot determine the effect of these SNPs on the development of drusen at a younger age. Although analysis of the Amish cohort has identified strong associations between *CFH*, *SYN3* and drusen area and volume, both false positive and false negatives may be present due to the overall small sample size. The stringency of our inclusion criteria, the use of a validated FDA-cleared OCT algorithm for drusen measurements and our reproducible genotyping are the strengths of our study. Our results also provide a foundation to further evaluate other SNPs in the known AMD genes for association with drusen phenotypes.

Color fundus photographs (CFPs) offer complementary information to SD-OCT [46]. Both methods play an important role in assessing drusen and RPE atrophy in patients with non-exudative AMD [47,48]. However, issues with the CFPs’ reproducibility due to fundus pigmentation variability and drusen appearance and media opacity negatively impacting photograph quality make SD-OCT a promising alternative for imaging drusen. SD-OCT provides information regarding drusen ultrastructure *in vivo* and quantifies the thickness of the drusen, as well as the photoreceptor layer above the drusen [49,50,51]. Future genetic correlation studies, however, may consider incorporating and integrating phenotypic information derived from multiple imaging modalities (e.g., color photographs, fundus autofluorescence, and OCT) to yield a more precise description of the disease phenotype.

## 5. Conclusions

Our data suggest that *CFH* and *SYN3* risk alleles play a role in determining the drusen phenotype in the Amish population. This approach of stratifying drusen phenotypes by genotype has the potential to separate AMD patients into homogenous groups for preventive and therapeutic studies. Of course, more SNPs will need to be evaluated to deliver such a genotype-phenotype classification.
